# Contrasting Experimentally Device-Manipulated and Device-Free Smiles

**DOI:** 10.3389/fpsyg.2019.02297

**Published:** 2019-10-15

**Authors:** Marie P. Cross, Liana Gheorma, Sarah D. Pressman

**Affiliations:** ^1^Department of Biobehavioral Health, The Pennsylvania State University, State College, PA, United States; ^2^Department of Psychology and Neuroscience, The University of North Carolina at Chapel Hill, Chapel Hill, NC, United States; ^3^Department of Psychological Science, University of California, Irvine, Irvine, CA, United States

**Keywords:** smiles, facial feedback hypothesis, facial EMG signals, facial expression, positive affect

## Abstract

Researchers in psychology have long been interested in not only studying smiles, but in examining the downstream effects of experimentally manipulated smiles. To experimentally manipulate smiles unobtrusively, participants typically hold devices (e.g., pens or chopsticks) in their mouths in a manner that activates the muscles involved in smiling. Surprisingly, despite decades of research using these methods, no study has tested to what degree these methods activate the same muscles as more natural, device-free smiles. Our study fills this gap in the literature by contrasting the magnitude of muscle activation in device-free smiles against the popular chopstick/pen manipulation. We also contrast these methods against the Smile Stick, a new device specifically designed to manipulate smiles in a comfortable and hygienic fashion. One hundred fifty-nine participants each participated in three facial expression manipulations that were held for 1 min: smile manipulation via Smile Stick, smile manipulation via chopsticks, and device-free smile. Facial electromyography was used to measure the intensity of the activation of the two main types of muscles involved in genuine, Duchenne smiling: the orbicularis oculi (a muscle group around the eyes) and the zygomaticus major (a muscle group in the cheeks). Furthermore, following each manipulation, participants rated their experience of the manipulation (i.e., comfort, fatigue, and difficulty), experienced affect (positive and negative), and levels of arousal. Results indicated that the Smile Stick and chopsticks performed equally across all measurements. Device-free smiles were rated as most comfortable but also the most fatiguing, and procured the greatest levels of positive affect and lowest levels of negative affect. Furthermore, device-free smiles resulted in significantly higher levels of both zygomaticus major (by ∼40%) and orbicularis oculi (by ∼15%) muscle activation than either the Smile Stick or chopsticks. The two devices were not different from each other in muscle activation. This study reveals that while device-free smiling procures the greatest changes in muscle activation and affect change, smiling muscle groups are activated by device manipulations, and expected changes in affect do occur, albeit to a lesser degree than device-free smiling. It also indicates that the Smile Stick is an acceptable and comparable alternative to disposable chopsticks.

## Introduction

Smiling has been a subject of fascination across a wide variety of fields for decades and has been investigated in areas such as mental health (e.g., VanSwearingen et al., 1999), physiology (e.g., Fredrickson and Levenson, 1998; Tsai et al., 2002), and social relationships (e.g., Mackey, 1976). Much of this research has focused on smiles that spontaneously occur (e.g., Fredrickson and Levenson, 1998; Davidson et al., 2010). However, another line of research is aimed at making causal inferences on experimentally manipulated smiles, or those that are covertly or overtly generated in the laboratory. To experimentally manipulate smiles unobtrusively, participants typically hold devices like pens or chopsticks horizontally in their mouths so that they activate the muscles involved in smiling. Experimentally device-manipulated smiles are important because, with a creative cover story (e.g., Tourangeau and Ellsworth, 1979; Strack et al., 1988), participants are typically not aware that they are smiling. This can decrease or even eliminate cognitive associations or demand characteristics that participants may have with smiles so the researchers can be confident that the psychological and physiological responses are indicative of facial muscle activation rather than some confounding variable.

While this research has been ongoing for decades, some important first steps have been skipped, and there are some striking open questions regarding this methodology. First, to the best of our knowledge, there is no existing study that examines whether the device-in-mouth style of facial manipulation activates the muscle groups involved in smiling. The extent to which smiling-related muscles are activated by device manipulations and how these levels of muscular activation compare to more natural, device-free smiles (i.e., with no device in the mouth) are completely unknown. This is critical missing information that prevents the ability to connect experimental device-manipulated smiling findings to real-world smiling.

This type of research is especially critical given the recent controversy in the area of facial feedback. The facial feedback hypothesis (FFH) posits that merely activating the facial muscles associated with specific emotions may be enough to elicit those emotions or influence the emotional experience (Tourangeau and Ellsworth, 1979). One of the first and most widely known studies to test this hypothesis had participants rate how amused they felt by a number of cartoons while they were holding a pen in their mouth to covertly induce either a smile or pout (Strack et al., 1988). Smiling participants found the cartoons to be funnier than those who were pouting, providing evidence for the FFH. However, this landmark study recently failed to replicate in a highly publicized pre-registered study conducted by 17 different labs (Wagenmakers et al., 2016). While many might take this as evidence against the FFH, a more critical takeaway is that we should try to understand *why* the FFH did not replicate and consider factors that may influence when the FFH applies or not (e.g., specifics of the independent variable and context). There are ample studies supporting the FFH in various contexts (e.g., VanSwearingen et al., 1999; Soussignan, 2002; Davis et al., 2010; Davies et al., 2011; Philippen et al., 2012), including a recent study of over 400 undergraduate students that used contemporary cartoons (Marsh et al., 2018). In addition, a recent meta-analysis of 136 facial feedback studies found an overall effect of facial feedback on affective experience (*d* = 0.20; Coles et al., 2019). Thus, it may be time to pay closer attention to this line of research, and, in particular, the relevance of two variables that have been ignored: strength and type of smile.

**FIGURE 1:**
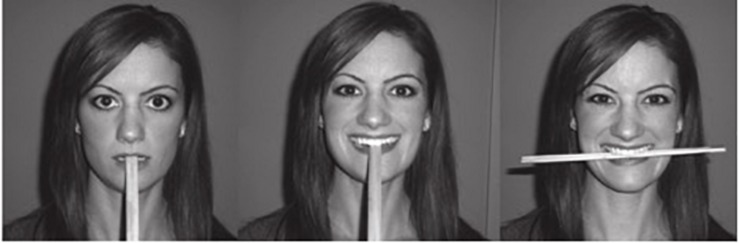
Chopsticks have been used in past literature in order to induce neutral facial expressions **(left panel)**, non-Duchenne smiles **(center panel)**, and Duchenne smiles **(right panel)**. This figure adopted from Kraft and Pressman (2012).

In some of the earliest work on the FFH, participants were told to make a specific facial expression (e.g., Laird, 1974). However, later work determined that this method was not ideal because it might activate cognitive expectancies associated with making specific facial expressions (e.g., Buck, 1980). A number of theorists have hypothesized that facial feedback can operate outside of cognitive awareness through direct physiological mechanisms that could generate affective reactions (e.g., Tomkins, 1962; Izard, 1977; Ekman et al., 1983). Therefore, in order to test this and covertly manipulate facial expression, participants in the seminal facial feedback study by Strack et al. (1988) held pens between their teeth in order to activate the zygomaticus major muscles (muscles in the cheeks that extend from each cheekbone to the corner of the mouth). This methodology has been common practice in countless FFH studies (see Coles et al., 2019, for examples). More recent studies had participants hold chopsticks in their mouths, since chopsticks are more easily disposable and hygienic than pens (see Figure 1; Kraft and Pressman, 2012; Pressman et al., under review). However, no study has objectively examined to what extent a device-free smile compares to a manipulated smile. The closest, albeit different, approach that tries to deal with the problem of comparability to real smiles has been simply coding for adherence to device smile condition on a “poor adherence” to “excellent adherence” scale (e.g., Kraft and Pressman, 2012) with hand coders who are trained in the Facial Action Coding System (Ekman and Friesen, 1978). While this is an improvement, it does not answer the question of degree of specific muscle activation since raters cannot see subtle differences in facial musculature. One other methodological concern that has been ignored is the issue of whether holding a device in the mouth in a manner that activates smile muscles is uncomfortable. Surprisingly, no one has asked participants to what extent this procedure is tiring, uncomfortable, or difficult, or whether these feelings are different from holding a self-created smile for the same period. If these manipulations are painful or uncomfortable, this could have critical implications for emotion-related outcomes, which are of greatest interest to FFH researchers. These questions have important implications for FFH and other smiling-related research study designs.

**FIGURE 2:**
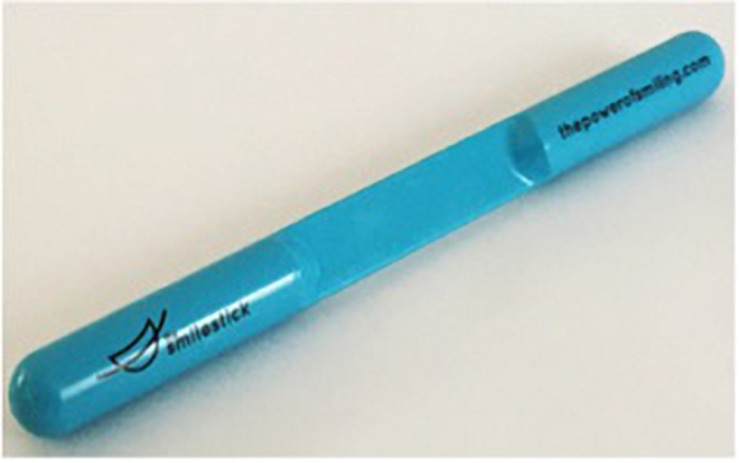
A Smile Stick.

Another concern of note within device-manipulated smile research is that neither pens nor chopsticks were designed to effectively manipulate smiles. One might wonder how past cognitive connections to these writing and eating tools might influence the effect of the device on outcomes of interest and/or whether they might actually be uncomfortable in some manner. With these issues in mind, a new device called the Smile Stick may be useful, which was crafted specifically to effectively and comfortably manipulate smiles. Smile Sticks are blue oral cylindrical devices with an indentation in the middle for correct teeth positioning. They are made out of Bayer Makrolon^®^, a non-toxic, FDA-approved material (see Figure 2), and are reusable as well as easily sanitized. Beyond comfort, this device may have the benefit of seeming more professional and hygienic in a research setting (since it is a medical device) as compared to other tools used in past research like pens and chopsticks that were not designed for this purpose, may not be easily sanitized, and may even have unanticipated problems like giving splinters (wooden chopsticks), being slippery to hold (plastic chopsticks), or leaking (pens). We test whether this new device is comparable to the past utilized chopsticks/pen approach as well as how it compares to device-free smiles. Importantly, should the Smile Stick be effective, future smile manipulation studies could use this more environmentally friendly and cost effective approach, as researchers would need only a few Smile Sticks that they could appropriately sterilize and reuse rather than purchase brand new pens or chopsticks for each participant in the study.

Thus, the primary goals of this study are to determine to what degree experimental smile manipulation studies are generating smiles as they occur in the real world, as well as to examine whether this methodology procures similar experiences and outcomes as device-free smiles. To examine this question, we test two forms of device-manipulated smiles against device-free smiles for the purpose of empirically examining the degree of difference in objective muscle activation, as well as to investigate some related variables (e.g., comfort, difficulty, muscle fatigue) that might influence future study design. The answers to these questions could have implications for the downstream emotion outcomes in which many researchers are interested. Furthermore, are there differences in the extent to which participants feel positive, negative, or aroused after each facial expression manipulation?

When deciding on instructions and methodology, we opted to focus on Duchenne smiles (i.e., those that activate both the zygomaticus major muscles in the cheeks and the orbicularis oculi muscles around the eyes). Surprisingly, past research on smiling manipulation has mostly ignored the non-Duchenne (i.e., smiles of non-enjoyment that only activate the zygomaticus major muscles in the cheeks) vs. Duchenne smile distinction and simply focus on having a device in the mouth, typically opting to induce only non-Duchenne smiles (see Kraft and Pressman, 2012, for an exception). This is surprising given that non-Duchenne smiles are not only poor inducers of positive feelings, but they may also increase negative outcomes (e.g., Goldberg and Grandey, 2007; Wagner et al., 2014). If the goal of FFH testing is to determine whether an expression of an emotion can induce or manipulate an emotion, Duchenne smiling is the expression that should be manipulated in the case of smiling since it is the smile type most tied to the experience of positive emotion (e.g., Ekman et al., 1990).

## Materials and Methods

### Participants

One hundred fifty-nine participants (73.5% female, *M*_age_ = 20.74) were recruited to participate in this study through the psychology human research subject pool at the University of California, Irvine, and via campus fliers. This sample size was based on a power analysis with power set at 0.9 and effect size set at 0.25 (medium effect size). Psychology subject pool participants received course credit. Flier participants were compensated $10 for the 1 h session. The sample was comprised of 45.9% Asian participants, 28.9% Hispanic/Latino participants, 13.2% White/Caucasian participants, 3.1% Biracial/Multiracial participants, 2.5% Black/African American participants, and 6.3% participants belonging to other ethnicities. The study was approved by the Institutional Review Board at the University of California, Irvine.

### Procedure

Participants came into the lab and were consented. Participants were told that the purpose of the study was to investigate the best way to activate facial muscles during three separate tasks. They then washed their faces in order to remove products that could affect electromyography (EMG) sensor recordings or adhesion (e.g., oils and lotions). Five EMG sensors were placed on the participants’ faces to measure facial muscle activation. Next, participants completed a number of baseline questionnaires on a computer, including demographics such as age, sex, and race/ethnicity. Following these questionnaires, participants sat still for 5 min while baseline EMG data were collected. All participants completed all three facial expression manipulations: chopsticks, Smile Stick, and device-free smile. The order of the device manipulations (chopsticks and Smile Stick) was randomized, but the device-free smile manipulation was always last to reduce participant awareness of the study goals.

**FIGURE 3:**
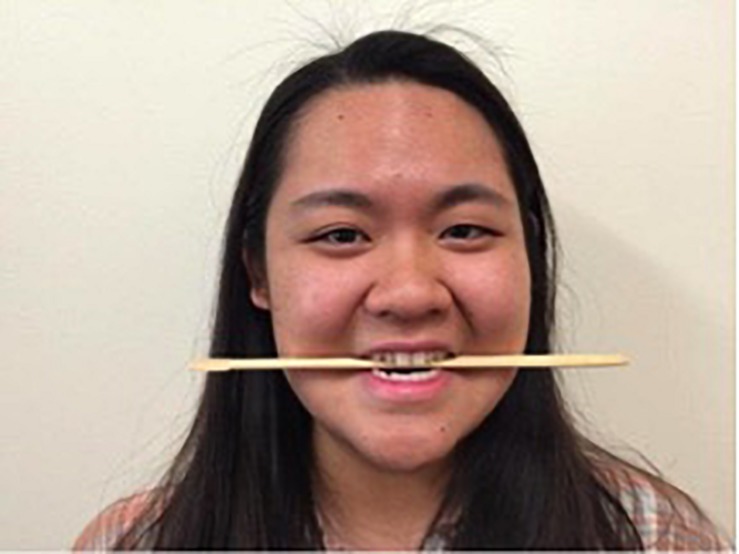
The example photograph of chopsticks that was shown to all participants (written informed consent was obtained from this individual for the publication of this image).

**FIGURE 4:**
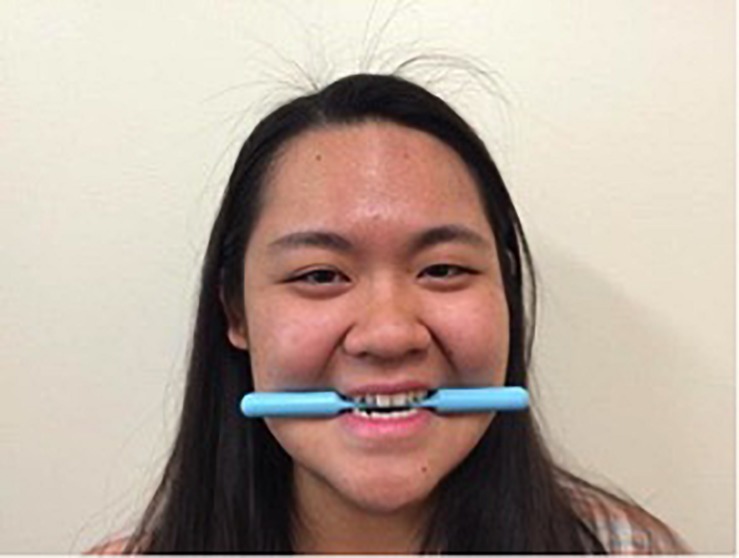
The example photograph of the Smile Stick that was shown to all participants (written informed consent was obtained from this individual for the publication of this image).

**FIGURE 5:**
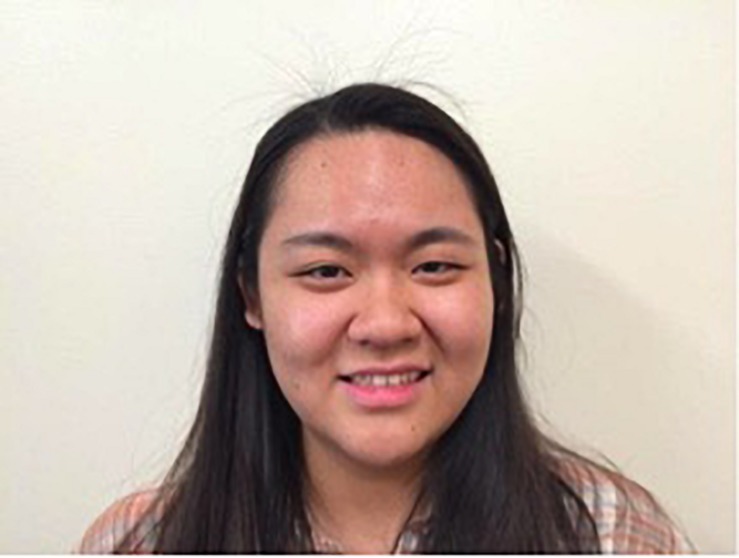
The example photograph of a device-free smile that was shown to all participants (written informed consent was obtained from this individual for the publication of this image).

Participants were trained in the correct technique for each manipulation for approximately 2 min immediately prior to each 1 min data collection period. For the chopsticks and Smile Stick manipulations, participants were shown photographs of someone holding either the chopsticks or Smile Stick in their mouth (see Figures 3, 4).^1^ Participants received the following instructions: “For this task, please place the (chopsticks/device) in your mouth, copying the person in the picture. Now, please hold (them/the device) in your mouth tightly. Make sure that your teeth are showing at all times. Be sure to mimic the facial actions exactly as they are shown in the picture.” For the device-free smile manipulation, participants were shown a photograph of someone displaying a Duchenne smile (see Figure 5). Participants received the following instructions: “For this final task, please smile naturally like the person in the picture. Please smile as big as you can and as sincerely as possible.” During the training periods for each manipulation, research assistants ensured that the participant’s teeth were showing and that their eye and cheek muscles were activated. Participants were corrected during the training period as necessary, but were not corrected during the 1 min data collection periods. Participants held each expression for 1 min while EMG was continuously assessed. After each manipulation, participants completed self-report measures of comfort, difficulty, and muscle fatigue and how positive, negative, and aroused they felt. Participants then moved on immediately to the training for the next manipulation. After all three manipulations were finished, participants were debriefed about the study.

### Measures

#### Self-Report

Immediately following each manipulation, participants were asked seven questions, each rated on a scale of 0–100. Participants were asked to rate their experiences with each of the three device positions on muscle fatigue (“How tired are your facial muscles?”), difficulty (“How difficult was it to hold the facial device like this?”), and comfort (“How comfortable was it to hold the facial device in this way?”). One question was aimed at determining how aroused participants felt [“How aroused/activated (i.e., energetic and aroused) do you feel right now?”], and one was aimed at determining how unaroused participants felt (“How unaroused/unactivated (i.e., tired and low energy) do you feel right now?”). Finally, participants reported on their positive and negative affect after the manipulation by answering, “How positive (excited, happy, and calm) do you feel right now?” and “How negative (angry, anxious, and sad) do you feel right now?”

#### Facial EMG

Facial EMG was used to continuously measure activation of the zygomaticus major and orbicularis oculi muscles. Five EMG sensors were applied to the left side of the participants’ faces as past research has shown that the left side of the face is more expressive (Mandal et al., 1995). The sensors were set to record at a sample rate of 500 Hz, gain at 2000 Hz, low cutoff at 20 Hz, and high cutoff at 200 Hz. EMG data were cleaned prior to analysis by removing the portions in which the participants made movements that interfered with facial muscles of interest, such as yawning or sneezing. EMG average activation for the two muscle groups of interest was exported for every 10 s of baseline and each 1 min trial, and then averaged to get an overall EMG average activation score for each time period of interest. Change scores used in analyses were calculated by subtracting average baseline muscle activation from muscle activation during each facial expression manipulation.

### Data Analyses

All data were analyzed with repeated measures analyses of variance in order to test differences within subjects across the three manipulations, including Bonferroni corrections, where appropriate. Mauchly’s Test of Sphericity was violated in all analyses; therefore, the Greenhouse-Geisser correction for violation of sphericity was used.

## Results

### Muscle Activation

#### Zygomaticus Major

**FIGURE 6:**
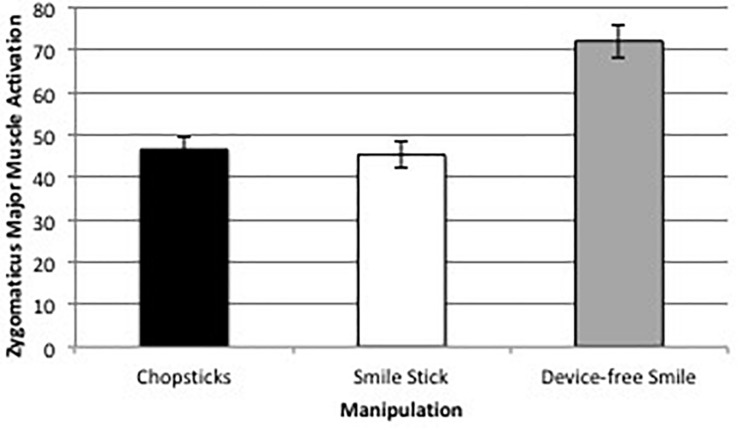
Average zygomaticus major muscle activation change (in uV) across the three facial expression manipulations.

The three manipulations led to significantly different levels of change in zygomaticus major muscle activation from baseline, *F*(1.29,191.80) = 47.43, *p* = 0.001, η*^2^_*partial*_* = 0.24. *Post hoc* comparisons using the Bonferroni correction revealed that zygomaticus major muscle activation was significantly higher during device-free smiles [*M* = 72.10 microvolts (uV), *SD* = 49.00 uV] than while holding the Smile Stick (*M* = 45.29 uV, *SD* = 38.26; *p* < 0.001) or chopsticks (*M* = 46.59 uV, *SD* = 36.14; *p* = 0.001), but there were no significant differences in zygomaticus major muscle activation between holding the Smile Stick and chopsticks (*p* = 1.0; see Figure 6). This amounted to the device-free smile manipulation having almost 40% more zygomaticus major muscle activation than either the Smile Stick or the chopsticks.

Zygomaticus major muscle activation was not significantly correlated with positive affect, negative affect, or feelings of arousal across any of the three manipulations (all *p*s > 0.05; see Tables 1–3).

**TABLE 1:** Correlation matrix for self-reported outcomes and facial muscle activation for the chopsticks manipulation.

	**Comfort**	**Muscle fatigue**	**Difficulty**	**Positive affect**	**Negative affect**	**Aroused**	**Unaroused**	**ZMM activation change score**	**OOM activation change score**
Comfort	1	−0.200^∗^	–0.317^∗∗^	0.286^∗∗^	–0.106	0.300^∗∗^	–0.199^∗∗^	–0.082	–0.080
Muscle fatigue	–	1	0.474^∗∗^	–0.122	0.419^∗∗^	–0.012	0.055	0.063	0.027
Difficulty	–	–	1	0.050	0.369^∗∗^	0.033	0.055	0.188^∗^	0.164^∗^
Positive affect	–	–	–	1	–0.141	0.500^∗∗^	–0.238^∗∗^	0.067	0.078
Negative affect	–	–	–	–	1	0.072	0.220^∗^	0.104	0.096
Aroused	–	–	–	–	–	1	–0.482^∗∗^	0.041	0.047
Unaroused	–	–	–	–	–	–	1	–0.079	–0.120
ZMM activation change score	–	–	–	–	–	–	–	1	0.614^∗∗^
OOM activation change score	–	–	–	–	–	–	–	–	1

*ZMM, zygomaticus major muscle; OOM, orbicularis oculi muscle. ^∗^*p* < 0.05, ^∗∗^*p* < 0.01.*

**TABLE 2:** Correlation matrix for self-reported outcomes and facial muscle activation for the Smile Stick manipulation.

	**Comfort**	**Muscle fatigue**	**Difficulty**	**Positive affect**	**Negative affect**	**Aroused**	**Unaroused**	**ZMM activation change score**	**OOM activation change score**
Comfort	1	−0.124	–0.389^∗∗^	0.305^∗∗^	–0.038	0.236^∗∗^	–0.137	–0.055	–0.062
Muscle fatigue	–	1	0.434^∗∗^	−0.166^∗^	0.426^∗∗^	–0.092	0.280^∗∗^	0.100	–0.012
Difficulty	–	–	1	–0.002	0.289^∗∗^	0.008	0.125	0.032	–0.019
Positive affect	–	–	–	1	–0.099	0.516^∗∗^	−0.194^∗^	0.037	0.080
Negative affect	–	–	–	–	1	0.085	0.293^∗∗^	0.080	0.084
Aroused	-	–	–	–	–	1	–0.392^∗∗^	0.130	0.074
Unaroused	–	–	–	–	– –	−	1	–0.112	–0.159
ZMM activation change score	–	–	–	–	–	–	–	1	0.661^∗∗^
OOM activation change score	–	–	–	–	–	–	–	–	1

*ZMM, zygomaticus major muscle; OOM, orbicularis oculi muscle. ^∗^*p* < 0.05, ^∗∗^*p* < 0.01.*

**TABLE 3:** Correlation matrix for self-reported outcomes and facial muscle activation for the device-free smile manipulation.

	**Comfort**	**Muscle fatigue**	**Difficulty**	**Positive affect**	**Negative affect**	**Aroused**	**Unaroused**	**ZMM activation change score**	**OOM activation change score**
Comfort	1	−0.172^∗^	–0.473^∗∗^	0.327^∗∗^	–0.083	0.163^∗^	–0.147	−0.176^∗^	–0.120
Muscle fatigue	–	1	0.436^∗∗^	–0.018	0.306^∗∗^	–0.003	0.257^∗∗^	0.176^∗^	0.044
Difficulty	–	–	1	–0.088	0.181	–0.054	0.231^∗∗^	0.092	–0.039
Positive affect	–	–	–	1	–0.133	0.655^∗∗^	−0.186^∗^	0.087	0.083
Negative affect	–	–	–	–	1	0.008	0.453^∗∗^	–0.014	–0.041
Aroused	–	–	–	–	–	1	–0.353^∗∗^	0.050	0.053
Unaroused	–	–	–	–	–	–	1	–0.114	−0.208^∗^
ZMM activation change score	–	–	–	–	–	–	–	1	0.544^∗∗^
OOM activation change score	–	–	–	–	–	–	–	–	1

*ZMM = zygomaticus major muscle; OOM = orbicularis oculi muscle. ^∗^*p* < 0.05, ^∗∗^*p* < 0.01.*

#### Orbicularis Oculi

**FIGURE 7:**
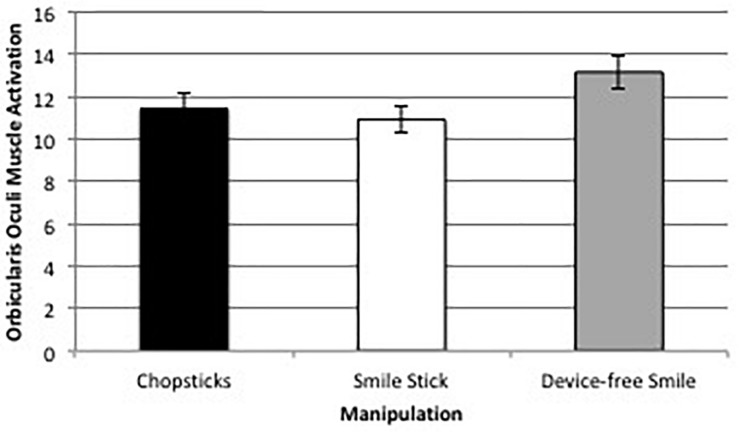
Average orbicularis oculi muscle activation change (in uV) across the three facial expression manipulations.

There was a significant difference between orbicularis oculi muscle activation change across the three manipulations, *F*(1.38,205.56) = 8.06, *p* = 0.002, η*^2^_*partial*_* = 0.05. *Post hoc* comparisons using the Bonferroni correction revealed that orbicularis oculi muscle activation was significantly higher during device-free smiles (*M* = 13.16 uV, *SD* = 10.08) than while holding the Smile Stick (*M* = 10.92 uV, *SD* = 7.99; *p* = 0.004) or chopsticks (*M* = 11.43 uV, *SD* = 8.93; *p* = 0.03), but there were no significant differences in orbicularis oculi muscle activation between holding the Smile Stick and chopsticks (*p* = 0.39; see Figure 7). This difference, while significant, was substantially smaller than the zygomaticus major difference, with only about a 15% difference between device-free smiles and either of the device manipulations.

Orbicularis oculi muscle activation was not significantly correlated with positive or negative affect across any of the three manipulations (all *p*s > 0.05). However, in the device-free smile manipulation, orbicularis oculi muscle activation was significantly negatively correlated with how unaroused participants felt following the manipulation, *r* = −0.208, *p* < 0.05.

### Self-Reported Facial Expression Experience

#### Difficulty

There were no significant differences in the self-reported level of difficulty between the three manipulations, *F*(1.73,245.14) = 3.04, *p* = 0.06, η*^2^_*partial*_* = 0.02. However, given that this effect was marginal, it is useful to note that holding a device-free smile was the most difficult of the three manipulations.

#### Muscle Fatigue

Muscle fatigue scores significantly differed between the Smile Stick, chopsticks, and device-free smiles, *F*(1.78,254.92) = 21.67, *p* < *0.001*, η*^2^_*partial*_* = 0.13. *Post hoc* comparisons using the Bonferroni correction revealed that muscle fatigue was significantly higher during device-free smiles (*M* = 39.90, *SD* = 25.57) than while holding the Smile Stick (*M* = 28.61, *SD* = 24.59; *p* < 0.001) or chopsticks (*M* = 28.99, *SD* = 22.77; *p* < 0.001), but there were no significant differences in muscle fatigue between holding the Smile Stick and chopsticks (*p* = 1.0).

#### Comfort

There was a significant difference between the Smile Stick, chopsticks, and device-free smiles in levels of self-reported comfort, *F*(1.86,267.23) = 9.37, *p* = 0.001, η*^2^_*partial*_* = 0.06. *Post hoc* comparisons using the Bonferroni correction revealed that comfort levels were significantly higher during device-free smiles (*M* = 44.73, *SD* = 28.75) than while holding the Smile Stick (*M* = 35.62, *SD* = 23.01; *p* = 0.001) or chopsticks (*M* = 36.03, *SD* = 24.65; *p* = 0.003), but there were no significant differences in comfort between holding the Smile Stick and chopsticks (*p* = 1.0).

### Emotional Experience Following Facial Expression Manipulations

#### Positive Affect

There was a significant difference in positive affect between the three manipulations, *F*(1.88,280.65) = 7.28, *p* = 0.001, η*^2^_*partial*_* = 0.05. *Post hoc* comparisons using a Bonferroni correction revealed that positive affect was significantly higher immediately following device-free smiles (*M* = 51.19, *SD* = 26.59) than the Smile Stick (*M* = 46.23, *SD* = 27.39; *p* = 0.005) or chopsticks (*M* = 46.62, *SD* = 26.87; *p* = 0.008), but there were no significant differences in positive affect immediately following the Smile Stick and chopsticks (*p* = 1.0).

#### Negative Affect

There was a significant difference in negative affect between the three manipulations, *F*(1.82,196.36) = 7.83, *p* = 0.001, η*^2^_*partial*_* = 0.07. *Post hoc* comparisons using Bonferroni corrections revealed negative affect was significantly higher immediately following holding chopsticks (*M* = 21.10, *SD* = 25.40) than holding device-free smiles (*M* = 16.25, *SD* = 24.46; *p* < 0.001). There were no significant differences between the Smile Stick (*M* = 18.87, *SD* = 24.39) and device-free smiles (*p* = 0.19) or between the Smile Stick and chopsticks (*p* = 0.12).

### Self-Reported Feelings of Arousal

There was a significant difference in how aroused participants felt immediately following each manipulation, *F*(1.77,258.37) = 22.05, *p* < 0.001, η*^2^_*partial*_* = 0.13. *Post hoc* comparisons using the Bonferroni correction revealed that participants felt significantly more aroused after device-free smiling (*M* = 44.42, *SD* = 23.66) than holding the Smile Stick (*M* = 36.87, *SD* = 22.44; *p* < 0.001) or the chopsticks (*M* = 37.35, *SD* = 22.91; *p* < 0.001), but there were no significant differences between holding the Smile Stick and chopsticks (*p* = 1.0). Similarly, there was a significant difference in how unaroused and unactivated participants felt immediately following each manipulation, *F*(1.89,279.85) = 33.41, *p* < 0.001, η*^2^_*partial*_* = 0.18. *Post hoc* comparisons using the Bonferroni correction revealed that participants felt the most unaroused after holding the Smile Stick (*M* = 49.88, *SD* = 24.82; *p* < 0.001) or chopsticks (*M* = 49.54, *SD* = 27.20; *p* < 0.001) vs. after device-free smiling (*M* = 37.77, *SD* = 27.68), but there were no significant differences between holding the Smile Stick and chopsticks (*p* = 1.0).

### Results With Transformed Data

All dependent variables were investigated for normality via skewness statistics. Results indicated that it was often the case that data were skewed for one (or more) of the manipulation outcomes, but not for all three of the manipulation outcomes. After log (base 10) transformation of all data and replication of the previous analyses, there were only a few changes of note. The first was that orbicularis oculi activation during the Smile Stick manipulation was significantly higher than orbicularis oculi activation during the chopsticks manipulation, *F*(2,296) = 339.12, *p* < 0.001. Comfort, *F*(1.86,254.34) = 2.62, *p* = 0.08, and positive affect *F*(2,284) = 2.80, *p* = 0.06, were no longer significantly different between the three manipulations. Finally, negative affect became significantly higher following the chopsticks manipulation than the Smile Stick manipulation, *F*(2,174) = 12.08, *p* < 0.001.

## Discussion

This study tested the differences in muscle activation, emotional experience, and general manipulation experience of different approaches to experimental smile manipulation. What is clear from the results, and critical to the current facial feedback literature utilizing device-guided manipulations, is that the difference of muscle activation during device-free vs. device-manipulated smiles is striking. Specifically, zygomaticus major muscle activation was almost 40% higher when participants smiled on command vs. when a device was utilized, and orbicularis oculi muscle activation was approximately 15% higher during the device-free vs. device-manipulated smiles. However, it is also notable that EMG muscle activation was above zero in the desired muscle groups during all facial expression manipulations. This indicates that participants in past FFH studies likely activated the muscle groups of interest in some way. That said, given the magnitude of differences, FFH studies that use devices to manipulate facial expressions need to consider that their manipulation most likely differs from device-free smiles. Furthermore, given the large number of studies utilizing no checks and balances to make sure that the correct muscles were activated, we have to wonder whether some past FFH studies are truly manipulating the muscles (and related emotions) that they think they are.

When considering the self-report findings from this study, all three manipulations were equally feasible in terms of participants’ ability to hold the position for 1 min and not significantly different in ratings of difficulty. That said, device-free smiling was reported to be approximately 10% more fatiguing vs. holding devices in the mouth (chopsticks or Smile Stick). However, despite being more fatiguing, device-free smiling was also rated as approximately 10% more comfortable than holding either chopsticks or a Smile Stick.

Affect data revealed that positive affect and feelings of energy were highest in the device-free smiling manipulation and approximately five points higher (on a 100 point scale) than the device holding groups. Unfortunately, due to the study design, it is not possible to determine whether this effect was due to facial feedback from Duchenne smiles or demand characteristics from hearing the word “smile.” The negative affect difference was smaller, with the chopsticks group reporting the highest levels of negative affect (vs. the device-free smile group, which was the lowest). While negative affect levels were low overall, in studies manipulating smiling, it is important for researchers to know that some approaches may result in affect outcomes that better match their design goals. In addition, given the larger positive affect findings in the non-device group, it would behoove researchers to explore how smiling can be manipulated without a device, but also, unlike in this study, ideally without having to tell individuals to smile. In addition, the Smile Stick group reported the least energy/most feelings of being tired as compared to the device-free smile group. One final important point to consider in the self-reported analyses is that in all comparisons discussed above, the Smile Stick and chopsticks did not significantly differ from one another (except in a single transformed analysis). This indicates that choice of device to manipulate smiling can be based more on hygiene, cost, environmental, and/or study design decisions.

What is the role of sex in these findings? There is a robust literature that suggests there are differences in smiling between men and women. For example, a meta-analysis of 162 studies determined that women are more likely to smile than men (LaFrance et al., 2003). The fact that women naturally smile more than men may mean that they feel more comfortable manipulating their face into a smile in an experimental setting. The majority of the sample in this study was female, and females did indeed drive the effects for many of the outcome variables of interest (analyses not shown). Future work in this area should specifically design studies in order to continue to investigate possible sex differences within the experimental smiling literature, especially given recent work showing that sex moderates the associations between facial expressions and health (Thompson et al., 2019). Another consideration is the role of ethnicity in these findings. More specifically, we wondered whether Asian participants would differ from participants of other races/ethnicities, since this was the race/ethnicity of the individual in the example photographs shown to participants. However, this was not the case; Asian participants did not differ from participants of any other race/ethnicity on any of the dependent variables of interest (analyses not shown). Taken together, these findings indicate that while some past work has found connections between race/ethnicity, sex, and emotion variables, in this study, race/ethnicity and sex were not important moderators of the found differences between manipulations.

Another key distinction to highlight in the context of this study is that the smiles participants made were manipulated or instructed Duchenne smiles as opposed to spontaneous Duchenne smiles. A large literature on both manipulated and spontaneous Duchenne smiles has supported the claim that they are connected with the experience of positive emotion (e.g., Ekman et al., 1990; Messinger et al., 2001; Soussignan, 2002). This literature, along with additional research on the FFH (e.g., Davis et al., 2010; Davies et al., 2011; Philippen et al., 2012), suggests the importance of investigating manipulated Duchenne smiles in order to determine whether the mere activation of the facial muscles involved in genuine expressions of positive emotion can alter or even create the experience of positive emotion. However, other research highlights the importance of smile dynamics and questions whether Duchenne smiles are actually reliable and valid indicators of positive emotional experiences (e.g., Krumhuber et al., 2007; Krumhuber and Manstead, 2009). Thus, research should continue to investigate the role of different types of smiles, both spontaneous and manipulated, within specific affective, social, and physiological contexts.

This study has a number of implications for researchers attempting to manipulate smiles experimentally. This is the first study to test the degree to which device-based manipulations activate the correct muscle groups involved with device-free smiling. These two device-based methods procure zygomaticus major activation to a great extent, as well as a small amount of orbicularis oculi activation. Few studies have measured orbicularis oculi muscle activation in the context of smiles, so it is difficult to make comparisons of what large or small amounts of activation of this muscle group actually look like. However, one study that included this measurement while participants looked at pictures of Duchenne smiles reported higher means than those we found in our device-based manipulations, suggesting that the levels of activation we found in our study are, indeed, small (Surakka and Hietanen, 1998). This means that facial feedback studies and other research that needs to manipulate smiling via non-obvious methods can continue to use this form of induction.

When it comes to picking a manipulation method, both the chopsticks and Smile Stick effectively and equally activate the muscles involved in smiling, although they do so to a lesser extent than device-free smiles. Although the Smile Stick has some advantages over chopsticks such as being more environmentally friendly, seeming more professional than a pair of chopsticks or a pen, and being cost effective, these devices result in similar amounts of facial muscle activation and similar ratings of muscle fatigue, difficulty, and comfort. Therefore, researchers can choose to use either device and be reasonably confident that their findings can be compared with research using the other device.

The more pressing implication of this work is that device-free smiles create substantially and significantly more zygomaticus major and orbicularis oculi muscle activation. Thus, for researchers looking for the highest levels of muscle activation in smiles, telling a participant to smile works best. Unfortunately, because of the possible demand characteristics and cognitive associations inherent with being told to smile, researchers may be hesitant to tell their participants to smile depending on the goals of their study. Although a recent meta-analysis of facial feedback effects found no differences across studies in which participants were aware vs. not aware of the facial expression they were making (Coles et al., 2019), an important future direction for this work is to develop alternative techniques to elicit larger and more natural smiles from participants without their knowledge. For example, an automated and gamified facial analysis computer program that encourages participants to activate certain muscles in their face through a reward system (e.g., they receive points if they correctly activate the muscles) may be one method in which to solve this issue. Another possible method is to use a face-specific electrical muscle stimulation machine (similar to the electrical probes used by the famous neurologist Duchenne de Boulogne to trigger facial muscle contractions) in order to fully activate a participant’s facial muscles without his or her awareness. New and creative methods such as these will enrich and potentially improve this literature by securing more powerful manipulations of smiling.

There are, however, a number of limitations to this study. One limitation is that facial expression manipulations were not fully randomized (device-free smile was always the third and final manipulation), so we could not control for order effects. In addition, this study was within-subjects; thus, we could not fully erase the possible spillover effects (e.g., muscle fatigue, positive/negative affect) from one manipulation to the next. Another limitation is that some of our dependent variables, such as fatigue and negative affect, were highly correlated. Although we felt it was important to investigate all of our dependent variables separately, future studies may wish to collapse some of these dependent variables into groups. Further, participants in this study were university-aged students, so these results are not generalizable to other age groups. We also do not have information regarding the field of study of our participants, although it is likely that many of them were studying psychology, since they were recruited from the Psychology subject pool. Thus, some of our participants might have been aware of research on the FFH, which could have influenced their affect ratings in the study. In addition, the presence of EMG leads on the face may have altered participant behavior in some way, although this is true of all studies utilizing this method and was true of all manipulations. Finally, participants were told to smile in one of the manipulations, which could have triggered cognitive associations with smiling and influenced self-report (due to demand or reactance).

Despite these limitations, this work answers the important question of whether and to what degree manipulated smiles activate the muscles involved in genuine, Duchenne smiling. We demonstrate that both chopsticks and the Smile Stick are effective manipulations to induce smiles, as evidenced by non-zero EMG activation of both the zygomaticus major and orbicularis oculi muscles, so researchers can make their manipulation choice based on additional factors like hygiene and sustainability.

## Data Availability Statement

The dataset for this study will not be made publicly available because there is additional data within this data set that the researchers plan to publish on in the near future. Requests to access the dataset should be directed to the corresponding author.

## Ethics Statement

This study was carried out in accordance with the recommendations of the Institutional Review Board at the University of California, Irvine, with written informed consent from all subjects. All subjects gave written informed consent in accordance with the Declaration of Helsinki. The protocol was approved by the Institutional Review Board.

## Author Contributions

All authors developed the study design and contributed to the manuscript. MC and LG prepared the study, collected the data, and analyzed the data.

## Conflict of Interest

The authors declare that the research was conducted in the absence of any commercial or financial relationships that could be construed as a potential conflict of interest.

## Footnotes

1 Five FACS-trained coders coded Figures 3–5 for AU 6 (cheek raiser and orbicularis oculi), AU 7 (lid tightener and orbicularis oculi), and AU 12 (lip corner puller and zygomaticus major). All five coders agreed that all of these action units were activated in Figures 4, 5; three out of five coders agreed that all of these action units were activated in Figure 3.
